# Effect of Silane Coupling Agent Treatment of Aggregates on Mortar Workability, Strength and Interfacial Microscopic Properties

**DOI:** 10.3390/ma16237458

**Published:** 2023-11-30

**Authors:** Chengyan Hou, Haibo Zhang

**Affiliations:** 1 School of Materials Science and Engineering, Henan Polytechnic University, Jiaozuo 454000, China; hcy807030@163.com; 2 Henan Key Laboratory of Materials on Deep-Earth Engineering, Jiaozuo 454003, China

**Keywords:** mortar, aggregate, wettability, silane coupling agents (SCAs), interfacial transition zone (ITZ)

## Abstract

In this study, 3-aminopropyltriethoxysilane (KH550) and vinyltrimethoxysilane (KH171) were used to modify aggregates and prepare aggregates with different surface wettability. The effect of silane coupling agents on aggregate surface properties was characterized through FT-IR, XPS, contact angles and aggregate water absorption. The influence of the aggregate’s surface wettability on the interface properties was discussed using MIP, SEM, BSE and nanoindentation, and then the influence mechanism of the interface microscopic properties on the macro-properties of mortar was revealed. The results showed that the type of silane has an intense impact on the surface properties of aggregates. KH550-modified aggregates increased the contact angle from 23.6° to 59°, while KH171-modified aggregates increased it from 23.6° to 91.6°. Silane-modified aggregates reduced the surface hydrophilicity, thereby reducing the water absorption and improving the mortar’s workability. However, KH550-modified aggregates exhibited a more effective enhancement of the mortar’s mechanical properties. Specifically, KH550-modified aggregates reduced the aggregate’s surface hydrophilicity, consequently alleviating the interfacial edge effect. This led to a 10% increase in the compressive strength of the mortar, an 11.6% reduction in the porosity, an 8.4% decrease in the interfacial porosity, and a 42.3% increase in the elastic modulus of the ITZ. Meanwhile, the cement matrix obtained a greater amount of water for cement hydration, resulting in an increased degree of hydration and an 18.5% increase in the elastic modulus of the cement matrix. The improvement in the ITZ between the modified aggregates and the cement matrix is considered to be one of the fundamental reasons for the enhancement of the mortar’s performance.

## 1. Introduction

Concrete is widely recognized as one of the most favored construction materials globally, owing to its affordability, easy accessibility of raw materials, straightforward manufacturing process, and commendable strength and durability. The composition of concrete primarily comprises aggregates, cementitious materials, and the interface transition zone (ITZ) [1,2,3]. This ITZ serves as a crucial link between the aggregates and cementitious materials. However, it is important to note that this particular zone is considered the weakest constituent within concrete, primarily due to its elevated porosity, high calcium–silica ratio, limited hydration particle content, and accumulation of calcium hydroxide and Ettringite crystals with an ordered arrangement [4,5,6,7]. Consequently, these factors significantly impact the overall strength of concrete.

In recent years, silane coupling agents (SCAs) have been proven to be excellent surface modifiers [8,9]. The silane functional groups in SCAs can chemically react with hydroxyl groups on the aggregate surface, forming chemical bonds or complexes. This chemical reaction allows the SCAs to firmly adhere to the aggregate surface and form a strong interfacial connection with the cementitious matrix [10,11,12,13,14,15]. The formation of chemical bonds increases the bond strength in the ITZ, enhancing its shear and tensile resistance. SCAs can also interact with the aggregate surface through physical adsorption. The non-polar part (such as the alkyl chain) in SCAs can interact with the non-polar region on the aggregate surface through van der Waals forces, forming a physical adsorption layer. The formation of this layer increases the friction and adhesion in the ITZ, improving the stability and durability of the interface. After treatment with SCAs, a dense and uniform coverage layer forms on the aggregate surface [16,17,18]. This layer can fill the micro-pores and cracks on the aggregate surface, improving the surface morphology of the aggregate and the contact status between particles, thus enhancing the density of the ITZ. However, SCAs can also improve the strong hydrophilic properties of the aggregate surface. The mechanism is that the hydroxyl groups on the hydrolyzed silane interact with the hydroxyl groups on the inorganic material surface, and the organic groups on the SCAs replace the hydroxyl groups on the inorganic material surface, thereby altering the properties of the inorganic material surface. Different types of organic groups in different coupling agents can give materials different surface wettability [19,20,21]. However, no one has explained the mechanism of how SCAs affect mortars and the ITZ by changing the surface wettability of the aggregate.

3-aminopropyltriethoxysilane (KH550) and vinyltrimethoxysilane (KH171) are structurally similar SCAs that can form stable siloxane bonds and amino functional groups on the surface of inorganic materials, thereby effectively modifying the surface of inorganic materials and imparting specific chemical properties. Zhang [8] modified graphene oxide with KH550, and the modified graphene oxide showed higher dispersion in a cement slurry compared to unmodified graphene oxide, promoting cement hydration and reducing the total porosity of cement-based materials. Sebaibi [22] treated fibers with 0.5% KH171, resulting in a 10-fold increase in pullout energy and a three-fold improvement in the bond strength with the cement matrix. Peng [19] used KH550 to treat aggregates, enhancing the interfacial bond strength between the aggregates and asphalt.

Maso proposed that during the mixing of concrete ingredients with water, a water film forms on the surfaces of all solid particles, including aggregate particles [23]. However, the thickness of this water film is related to the hydrophilicity of the solid particles’ surface. Typically, the more hydrophilic the solid particle’s surface, the easier it is for water to spread and adsorb on its surface, resulting in a thicker water film. When hydrophilic aggregates are incorporated into concrete, they tend to adsorb more water in the fresh mixture, thereby increasing the moisture content in the ITZ. Meanwhile, under the influence of the edge effect, this leads to structural characteristics such as a higher porosity, an enrichment of calcium hydroxide and ettringite crystals, and a higher calcium-to-silica ratio, which subsequently affect the strength and durability of concrete. Therefore, it is of great significance to improve the microstructure of the ITZ by reducing the hydrophilicity of the aggregate surface.

Therefore, in this study, 3-aminopropyltriethoxysilane and vinyltrimethoxysilane were used to modify natural aggregates. The effects of these two different coupling agents on the surface properties of aggregates and the mechanical properties of mortar, as well as their influence on the microstructure and mechanical properties of the ITZ, were compared to elucidate the impact of aggregate surface wettability on mortar and the ITZ.

## 2. Experiments

### 2.1. Materials

The P·I42.5 cement used in this study was purchased from Fushun Ausail Science and Technology Co., Ltd. Fushun, China, and its chemical composition is shown in Table 1.

**Figure 1:**
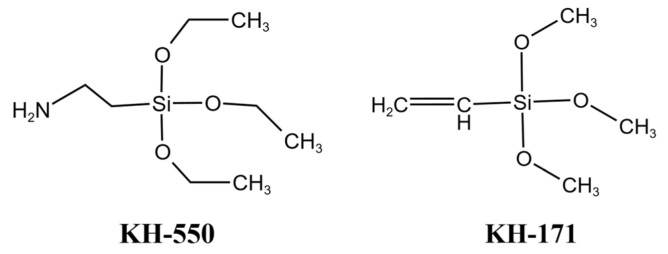
Molecular structures of KH550 and KH171.

The two types of coupling agents used in this study were 3-aminopropyltriethoxysilane (KH550) and vinyltrimethoxysilane (KH171). KH550 and KH171 were purchased from Shanghai Aladdin Bio-Chem Technology Co., Ltd., Shanghai City, China. The basic physical properties are presented in Table 2 and Table 3, and the chemical structures are shown in Figure 1.

The fine aggregate mainly consists of quartz sand (SiO_2_), with a maximum particle size of 4.75 mm and a fineness modulus of 2.7.

### 2.2. Surface Modification of Aggregates

Firstly, different types of SCA hydrolysates were prepared. The mass of each SCA and that of ethanol and deionized water is shown in Table 4.

The hydrolysis conditions of each hydrolysate are presented in Table 5.

**Figure 2:**
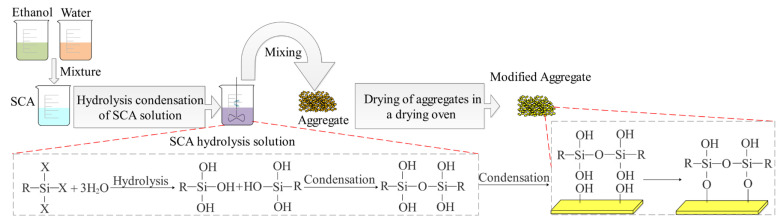
Modification process on the surface of aggregates.

After hydrolysis, the hydrolysates were mixed with the aggregates at 30 °C for 20 min. Then, the modified aggregates were placed in a 100 °C oven and baked for a specific time according to Table 5. After baking, the modified aggregates were taken out and allowed to cool down before preparing mortar samples. The specific modification process is shown in Figure 2.

To better illustrate the issue, the untreated aggregates and mortar were named as Un-FAs. The aggregates and mortar treated with KH550 were named KH550-FAs, and the ones treated with KH171 were named KH171-FAs.

### 2.3. Preparation and Curing of Mortar

The mortar was prepared by mixing water and cement in a ratio of 0.5 and cement and sand in a weight ratio of 1:3. The fresh mortar was poured into a mold measuring 40 mm × 40 mm × 160 mm and allowed to cure under standard conditions (humidity > 95%, temperature = 20 °C) for 1 day. Afterward, the specimens were removed from the molds, and we continued curing them under standard conditions until the desired test age (3 days, 7 days, 28 days) for conducting relevant performance tests.

### 2.4. Test Methods

#### 2.4.1. X-ray Diffraction (XRD)

The mineral composition of the aggregates before and after modification was measured using an X-ray diffractometer (Smart Lab, X-ray Diffraction, Tokyo, Japan). The sample preparation method is as follows: Before modification, the aggregates were crushed into powder using a crusher. Then, the obtained powder was modified according to the procedure outlined in Section 2.2. Finally, the modified aggregate powder was subjected to XRD testing. The equipment employed Cu Ka radiation (k = 0.154 nm). The voltage was set at 40 kV, and the current was set at 150 mA. The testing range and speed were set at 5–85° and 10°/min, respectively.

#### 2.4.2. Fourier Transform Infrared Spectroscopy (FT-IR)

The sample structures were characterized using a PerkinElmer Frontier FT-IR spectrometer (Waltham, MA, USA). The aggregates were crushed and sieved through a 200-mesh sieve for subsequent use. The sieved aggregates were modified according to the modification method outlined in Section 2.2. The modified aggregates were then washed multiple times with deionized water and ethanol to eliminate the effects of physical adsorption. Finally, they were dried in an oven at 100 °C. KBr powder and the samples were pressed into pellets, and the signals were accumulated through 16 scans. The wavenumber range was set between 4000 and 400 cm^−1^.

#### 2.4.3. X-ray Photoelectron Spectroscopy (XPS)

The chemical composition of the aggregate surface before and after modification was analyzed using an X-ray photoelectron spectrometer (K-Alpha, Thermo Scientific, Waltham, MA, USA). During the testing, an Al Kα X-ray source was employed, and the voltage was set to 12 kV.

#### 2.4.4. Contact Angle

The contact angle of the aggregate surface, which characterizes the wettability of the aggregate surface, was measured using a contact angle measurement instrument (JC2000D1, Shanghai Zhongchen Digital Technology Equipment Co., Ltd., Shanghai, China). The solid surface contact angle is influenced by the surface roughness. To eliminate the influence of the surface roughness on the contact angle, the aggregates were polished with 80-mesh sandpaper, and the roughness was measured to ensure consistent surface roughness of the aggregates. The aggregates were then cleaned using ultrasonic cleaning and dried for surface modification.

The cleaned aggregates were modified according to the procedure outlined in Section 2.2, and the contact angles before and after modification were measured. Deionized water was chosen to measure the contact angle on the aggregate surface. Each sample was measured six times, and the average value was taken as the final measurement result [24]. The modified aggregates were washed multiple times with deionized water and ethanol to eliminate the effects of physical adsorption. Finally, they were dried in an oven at 100 °C. After cooling, contact angle measurements were performed again.

#### 2.4.5. Fluidity

According to the Chinese National Standard GB/T 2419-2005 [25], the variation in mortar fluidity before and after modification was measured. The diameters of the freshly mixed mortar were measured using a ruler in two mutually perpendicular directions, and the average value was taken as the final result.

#### 2.4.6. Mercury Intrusion Porosimeter (MIP)

An automated mercury intrusion porosimeter instrument (9510, Micromeritics Instrument Corporation, Norcross, GA, USA) was utilized to perform pore structure testing on 28-day mortar samples. The impact of different types of coupling agents on the pore structure of the mortar samples was analyzed.

#### 2.4.7. SEM and Backscattered Electron Imaging (BSE)

The ITZ of the 28-day mortar was characterized using the secondary electron mode of a scanning electron microscope (Merlin Compact, Carl Zeiss NTS GmbH, Oberkochen, Germany). The relationship between interfacial porosity, hydration products, and unhydrated products with respect to the distance from the aggregate surface was measured using the backscattered electron mode. The backscattered signal is not only related to the atomic number but also correlated with the surface morphology of the material. To eliminate the influence of the surface morphology on the measurement results, the samples need to undergo polishing [26,27]. The specific procedure is as follows: firstly, a core sample was obtained by cutting a 28-day sample, resulting in a cubic sample with dimensions of 1 cm × 1 cm × 1 cm. Then, the cut sample was soaked in ethanol to halt hydration and subsequently dried in a drying oven at 50 °C. The dried sample was cold-embedded with low-viscosity epoxy resin. Next, the cured samples were polished sequentially using 400-grit, 800-grit, 1200-grit, 2000-grit and 3000-grit silicon carbide sandpaper for 5 min each. Subsequently, fine polishing was conducted for 15 min using polishing fluid with particle sizes of 3 μm and 0.5 μm. After each polishing step, the sample was subjected to ultrasonic cleaning to remove surface impurities.

#### 2.4.8. Nanoindentation

The elastic modulus of the ITZ in the mortar samples was determined using a nanomechanical indentation testing instrument (TL-Premier, Hysitron, Minneapolis, MN, USA). The sample preparation process was the same as described in the BSE section. The procedure for measuring the elastic modulus of the interface transition zone is as follows: 8 lines with a spacing of 5 μm and a length of 120 μm were selected for nanomechanical indentation testing in the direction perpendicular to the ITZ. The spacing between adjacent test points was 5 μm. The experimental load was set at 2000 μN, with loading and unloading times of 5 s and a holding time at the peak load of 2 s [28].

#### 2.4.9. Mechanical Properties

Based on the Chinese standard GB/T 17671-2021 [29], a computer-controlled flexural and compressive testing machine (YAW-300C, Jinan Liangong Testing Technology Co., Ltd., Jinan, China) was used to conduct tests on the compressive strength and flexural strength of the mortar samples. The mechanical properties of the mortar samples were tested at 3 days, 7 days, and 28 days. The flexural strength was measured three times for each age period, whereas the compressive strength was measured six times. The average values obtained from these measurements were considered the final results.

## 3. Results and Discussion

### 3.1. XRD

**Figure 3:**
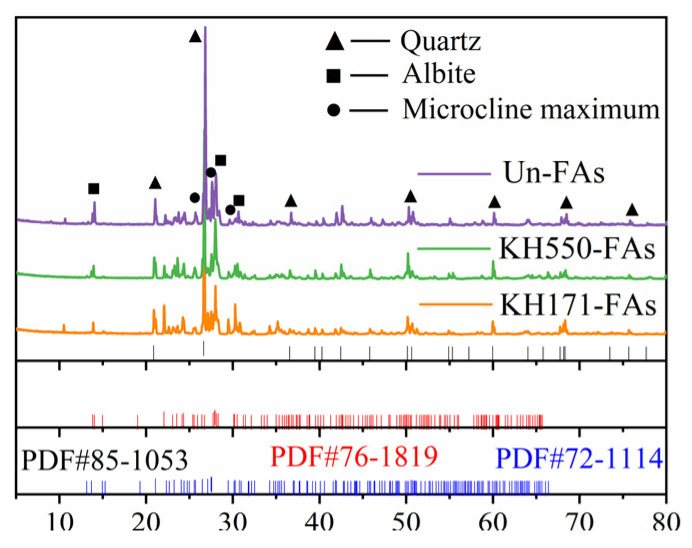
The XRD patterns of the aggregates before and after modification.

Figure 3 shows the XRD patterns of the aggregates before and after modification.

In the XRD pattern of the Un-FAs, the main peaks (2θ) are located at 20.8°, 26.6°, 36.5°, 50.1°, 55.3°, 59.9° and 68.3°, corresponding to the (100), (011), (110), (112), (013), (121) and (031) crystal planes of Quartz, respectively. The main peaks (2θ) are located at 13.8°, 27.9° and 30.2°, corresponding to the (020), (002), and (−2−22) crystal planes of Albite, respectively. The main peaks (2θ) are located at 25.5°, 27.4° and 29.5°, corresponding to the (−1−12), (−220) and (131) crystal planes of Microcline maximum, respectively. The XRD patterns of the modified aggregates are consistent with those of the unmodified aggregates, and no other phases were detected. The results indicate that the crystal structure of the aggregates remains unchanged before and after modification with the coupling agent.

### 3.2. FT-IR

**Figure 4:**
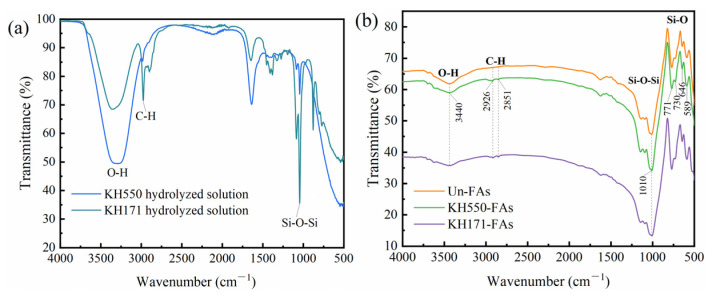
Infrared spectra of (**a**) silane coupling agent hydrolysate and (**b**) aggregates.

The modification effect of the coupling agent on the aggregates was investigated using Fourier-transform infrared spectroscopy. Figure 4 shows the infrared spectra of the hydrolysis products of the two coupling agents and the aggregates before and after modification.

Observing the infrared spectra of the aggregates in Figure 4b, the peak at 1010 cm^−1^ corresponds to the antisymmetric stretching vibration of Si-O-Si, and the peaks at 771 cm^−1^, 730 cm^−1^, 646 cm^−1^ and 589 cm^−1^ correspond to the symmetric stretching vibration of Si-O, which are typical characteristic peaks of quartz sand [30]. In the infrared spectra of the modified aggregate, new characteristic peaks appeared at 2926 cm^−1^ and 2851 cm^−1^, corresponding to the CH_2_ and CH groups, respectively [31], indicating that the SCAs or the hydrolysate of the SCAs successfully covered the surface of the aggregate. After the modification of the aggregates, an enhancement of the Si-O-Si absorption peak at 1010 cm^−1^ is observed, which may be attributed to the antisymmetric stretching vibration of the newly formed Si-O-Si bonds [10]. Meanwhile, a decrease in the stretching vibration peak of O-H at 3440 cm^−1^ is observed after the modification of the aggregates [32], indicating the replacement of hydroxyl groups on the aggregate surface by the hydrolysis products of the SCAs, resulting in a decrease in the number of hydroxyl groups on the aggregate surface. These results demonstrate that the coupling agent is successfully grafted onto the surface of the aggregates after the modification.

### 3.3. XPS

**Figure 5:**
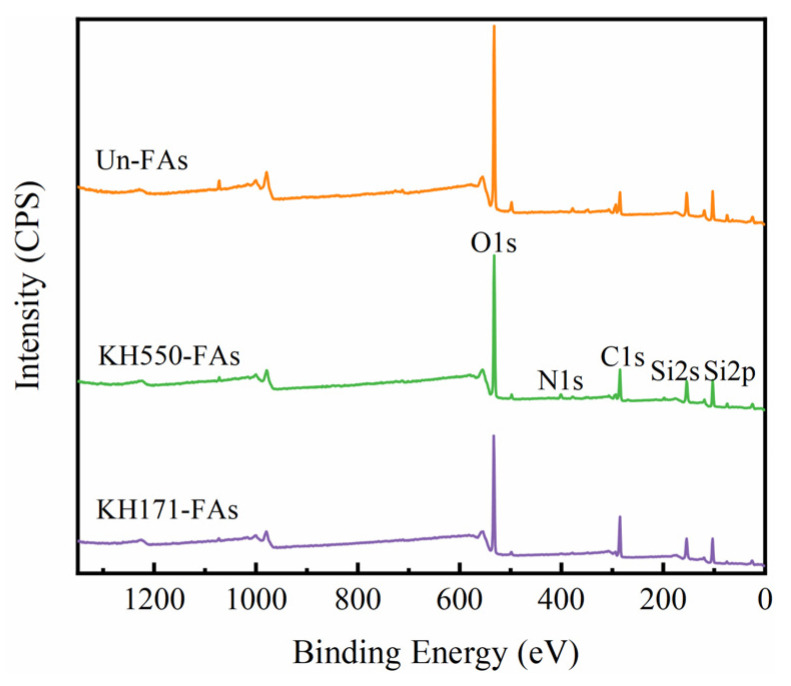
XPS spectra of aggregate surface before and after modified with SCAs.

The detection depth of XPS is only 5–10 nm for the material surface [17,33]. Therefore, XPS was used to analyze the chemical information on the material surface. The XPS analysis results before and after the modification of the aggregates are shown in Figure 5.

Table 6 describes the corresponding detailed information for Figure 5.

Observing Figure 5, two strong absorption peaks appear at 103 eV and 532 eV, indicating that the surface of the untreated aggregates mainly contains oxygen and silicon elements. Compared to the Un-FAs, a nitrogen element appears on the surface of the KH550-FAs, which is only related to KH550. Observing Table 6, the content of oxygen and silicon elements on the modified aggregate surface decreases significantly, while the carbon element content increases noticeably. This is because the carbon content in the coupling agent is higher than that in the aggregates [17,34]. All the results demonstrate the good interaction between the coupling agent and the aggregate surface.

### 3.4. Contact Angle of Aggregate Surface

**Figure 6:**
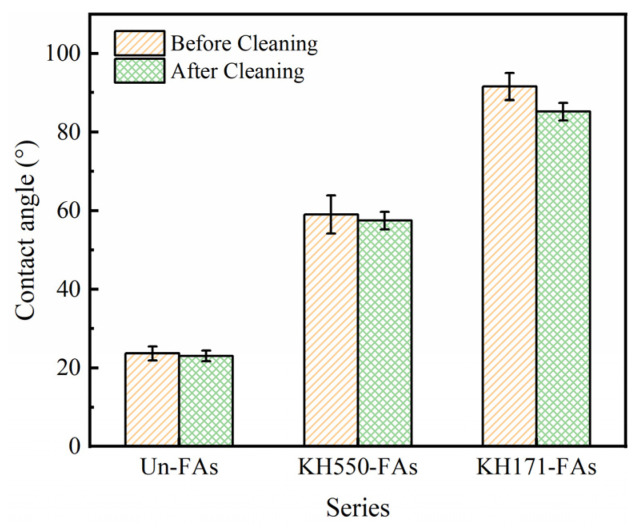
Contact angle of the aggregate surface modified by SCAs.

The wettability of the aggregate surface before and after the modification was characterized by contact angle test results. Figure 6 shows the influence of different coupling agents on the contact angle of the aggregate surface.

Observing Figure 6, the contact angles of the Un-FAs, KH550-FAs and KH171-FAs were 23°, 59° and 91°, respectively. The contact angle of the Un-FAs is 23°, which is due to the coverage of hydroxyl groups with strong hydrophilicity on the aggregate surface, resulting in a smaller contact angle. After modifying the aggregates with KH550, the amino groups in KH550 replaced the hydroxyl groups on the aggregate surface. Amino groups have lower hydrophilicity than hydroxyl groups. Therefore, after modifying the aggregates with KH550, the contact angle of the aggregate surface increases. However, after modifying the aggregates with KH171, the aggregate surface becomes hydrophobic. This phenomenon is attributed to the vinyl groups in KH171 replacing the hydroxyl groups on the aggregate surface, and vinyl groups exhibit strong repulsion when in contact with strongly polar water molecules [35]. Hence, the aggregate surface exhibits hydrophobicity. All the results indicate that the use of SCAs modifies the properties of the aggregate surface. After being rinsed with deionized water and ethanol, the change in contact angle of the modified aggregate surface is minimal, indicating that the coupling agent chemically adsorbs onto the aggregate surface. This conclusion is consistent with the findings from the FTIR and XPS analyses.

### 3.5. Water Absorption Rate of Aggregates

The prescribed numbers of Un-FAs, KH550-FAs, and KH171-FA were placed in a 100 °C oven for 12 h to remove trace moisture. After vacuum cooling at room temperature, the aggregates were weighed, and the initial mass was recorded as $w_{0}$. The aggregates were then immersed in water for 24 h, followed by weighing, and the mass was recorded as $w_{1}$. The water absorption rate [17] is expressed as follows: $X_{W}=\frac{W_{1}-W_{0}}{W_{0}}\times 100\%$. Three measurements were performed for each group, and the average value was taken as the final test result.

**Figure 7:**
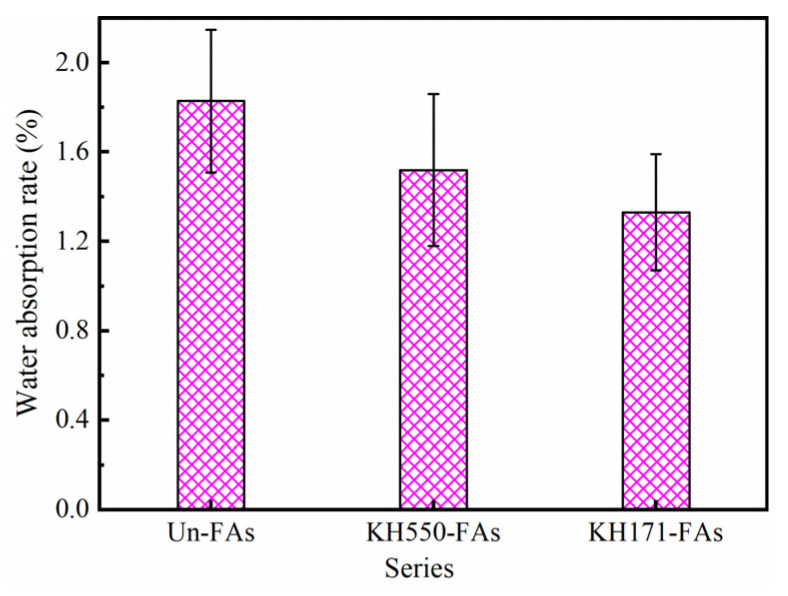
Water absorption of aggregates treated with SCAs.

Figure 7 presents the water absorption rate of the aggregates modified with different SCAs.

As observed in Figure 7, compared to the Un-FAs, it is evident that the water absorption rate decreases gradually for the KH550-FAs and KH171-FAs. The water absorption of the KH550-FAs decreased by 16.9%, and that of the KH171-FAs decreased by 27.3%. This can be attributed to the successful encapsulation of the coupling agent on the aggregate surface during the modification with SCAs [36]. At the same time, due to the increase in the contact angle, the hydrophobic property of the aggregate surface is enhanced. In particular, after the modification of KH171, the contact angle of the aggregate surface is greater than 90°, which changes from hydrophilic to lipophilic, resulting in decreased water absorption of the aggregate surface.

### 3.6. Fluidity of Mortar

**Figure 8:**
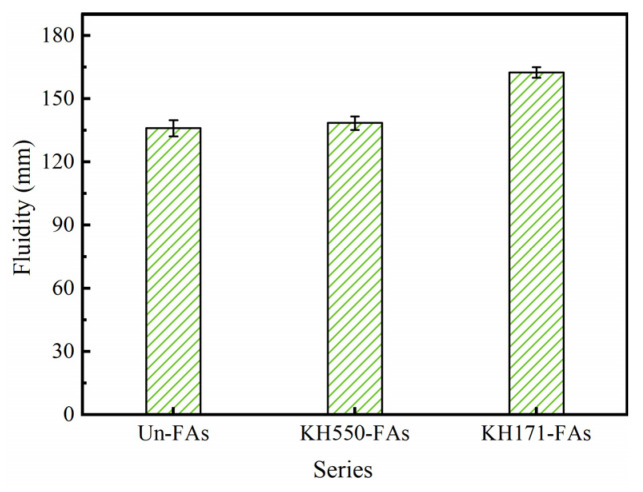
Fluidity of mortar before and after aggregate modification.

Figure 8 illustrates the fluidity of the mortar prepared before and after the aggregate modification.

Compared to the Un-FAs, both the KH550-FAs and KH171-FAs exhibit higher fluidity. Specifically, the fluidity of the KH171-FAs increased by 19.5% compared to the Un-FAs. However, no segregation or bleeding phenomena were observed in either case. These results indicate that the modification of aggregates with SCAs improves the workability of mortar. The primary reason for the increased workability is the significant reduction in the water absorption rate of the aggregates after the treatment with KH550. Consequently, the water absorption rate of the aggregates in the freshly mixed mortar is decreased, increasing the volume of water available for the normal hydration of the cementitious matrix and thereby improving the flowability of the mortar. However, when the aggregate is treated with KH171, the surface of the aggregate becomes hydrophobic. Due to the hydrophobic nature of the aggregate surface, when the aggregate is added to the newly mixed slurry, air is introduced into the slurry [37,38]. At the same time, under the action of vibration, some air enters the gelling matrix, thus providing a larger space for the flow of cement slurry to increase the fluidity of the mortar [39].

### 3.7. Porosity of Mortar

**Figure 9:**
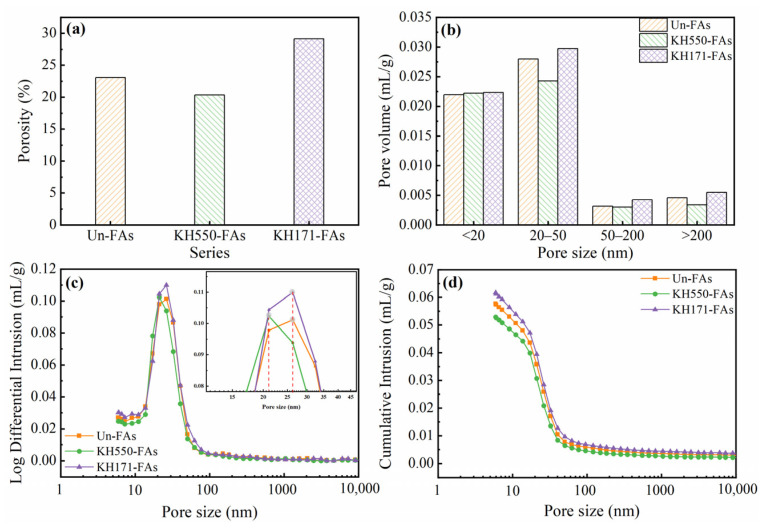
Pore structure of mortar at 28 days: (**a**) porosity; (**b**,**c**) pore size distribution; (**d**) cumulative pore volume.

Figure 9 depicts the pore structure of the mortar at 28 days.

The pore size distribution of the mortar can be classified into four types: harmless pores (≤20 nm), less harmful pores (20–50 nm), harmful pores (50–200 nm), and larger harmful pores (≥200 nm) [40]. Observing Figure 9a,b, compared to the Un-FAs, the KH550-FAs exhibited an 11.90% reduction in porosity, while the KH171-FAs showed a 26.07% increase in porosity. Additionally, the use of KH550-modified aggregates optimized the harmful pore structure of the mortar and further refined the pore size. The most probable pore size decreased from 26 nm to 21 nm (Figure 9c). This is because the water absorption rate of the aggregates decreases after treatment with KH550. As a result, the cementitious matrix receives more water for hydration, leading to an improved density of the mortar. However, the modification with the KH171 aggregate not only increased the overall pore volume of the mortar but also increased the volume of harmful pores, which has an adverse effect on the mortar. This is attributed to the hydrophobicity of the aggregate surface after treatment with KH171. Similar to rubber aggregates, when added to mortar, the hydrophobicity of the aggregate surface induces air entrainment. Some of these air voids exist at the interface between the aggregate and the matrix, while others are internal voids within the mortar matrix. Together, they contribute to an increased overall porosity of the mortar. Thus, the increase in harmful pores is also a result of this phenomenon [41,42]. Figure 9c shows that the peak of the pore distribution curve in the modified aggregate mortar is around 50 nm, which is the same as the peak of the pore distribution curve in the untreated mortar. Meanwhile, by observing Figure 9d, it is found that the three curves exhibit consistent trends, indicating that the modification does not alter the pore size distribution structure of the mortar [43].

### 3.8. Microstructure of the Interface Transition Zone in Mortar

**Figure 10:**
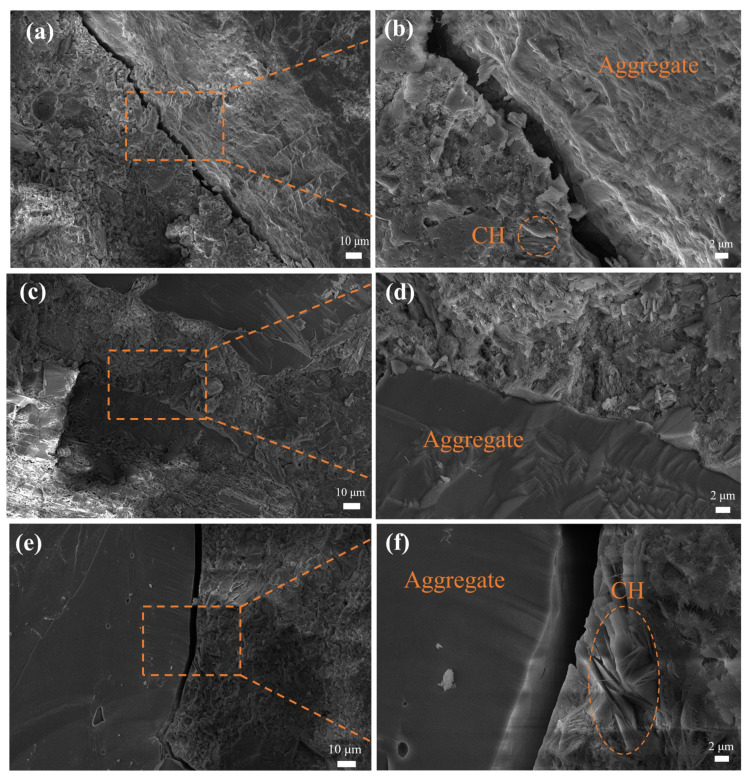
Microstructure of the ITZ in mortar: (**a**,**b**) Un-FAs; (**c**,**d**) KH550-FAs; (**e**,**f**) KH171-FAs.

Figure 10 illustrates the microstructure of the mortar interface before and after the aggregate modification.

From Figure 10a,b, it can be observed that there are significant cracks in the interface of the Un-FAs, accompanied by the phenomenon of an oriented arrangement of calcium hydroxide crystals. This is because the aggregate surface in the freshly mixed mortar is surrounded by a layer of water film, leading to a higher water–cement ratio near the aggregate compared to the water–cement ratio in the cementitious matrix [44]. As a result, the calcium hydroxide crystals become coarse with a higher porosity. These conditions provide the main locations for crack initiation and propagation, resulting in poor bond strength at the Un-FAs’ interface [45]. However, the use of KH550-modified aggregates effectively improves the bond performance at the mortar interface. This is primarily due to the increased contact angle between the modified aggregate surface and water, which enhances the driving force for water to leave the aggregate surface due to surface tension. Consequently, the water–cement ratio near the aggregate is reduced, restricting the growth space of the calcium hydroxide crystals, reducing the interface porosity, and improving the interfacial bond strength [46]. On the other hand, at the interface of the KH171-FAs, cracking is observed along with larger calcium hydroxide crystals. This can be attributed to two main reasons. Firstly, after being treated with KH171, the aggregate surface becomes hydrophobic with a lower surface energy. However, the cementitious matrix is typically a porous material with a higher surface energy, resulting in a weak mutual attraction between them [42]. This leads to poor bond performance between the cementitious matrix and the hydrophobic aggregate surface. Secondly, when hydrophobic aggregates are added to the cementitious matrix, water cannot spread completely on the aggregate surface due to its hydrophobicity. Instead, it introduces bubbles or forms local water voids on the aggregate surface. The formation of these local water voids allows the calcium hydroxide crystals generated from cement hydration to grow freely with oriented growth.

### 3.9. Backscattered Electron Imaging (BSE)

**Figure 11:**
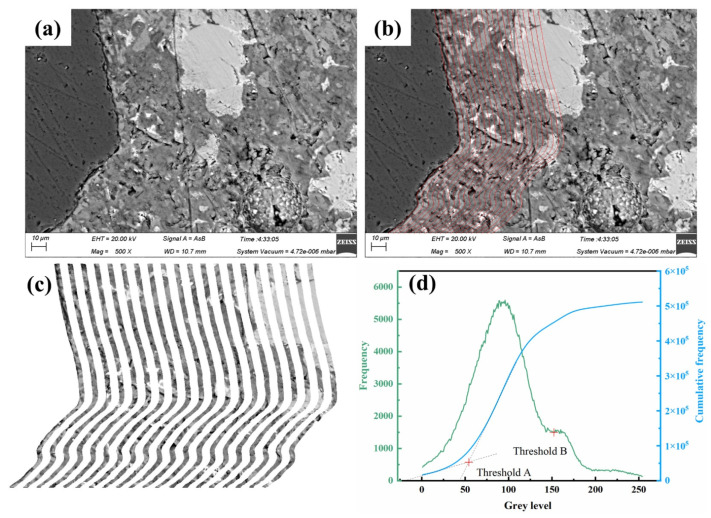
Illustration of the BSE image analysis process: (**a**) original image; (**b**) boundary capture; (**c**) strip division; (**d**) threshold determination.

The BSE image of the mortar interfacial transition zone is shown in Figure 11.

The BSE signal production is related to the average atomic number and surface morphology and thus can be used to differentiate phases in the material based on grayscale values. From dark to bright, the phases are, respectively, pores and cracks, hydration products, and unhydrated cement clinker particles [47]. By using the method described by Wang [48,49] to determine the grayscale range of each phase, the grayscale distribution of each phase in different regions at varying distances from the aggregate was quantified using a two-dimensional image analysis, as shown in Figure 11d. As not all samples were captured continuously, there may be variations in the sample grayscales, and therefore it was necessary to reconfirm the grayscale range of each phase.

Since mortar is a heterogeneous material, it is necessary to measure multiple areas to accurately reflect the actual situation. Therefore, 25 images [50] were captured for each sample at a magnification of 500. Each photo had a resolution of 1024 × 768 pixels. Subsequently, each image was sliced into continuous strips with a width of 3 μm, starting from the aggregate surface. The volume fraction of each phase was measured using the Image-pro-plus 6.0 image processing software based on the corresponding grayscale range belonging to each phase. The specific operational process is shown in Figure 11.

**Figure 12:**
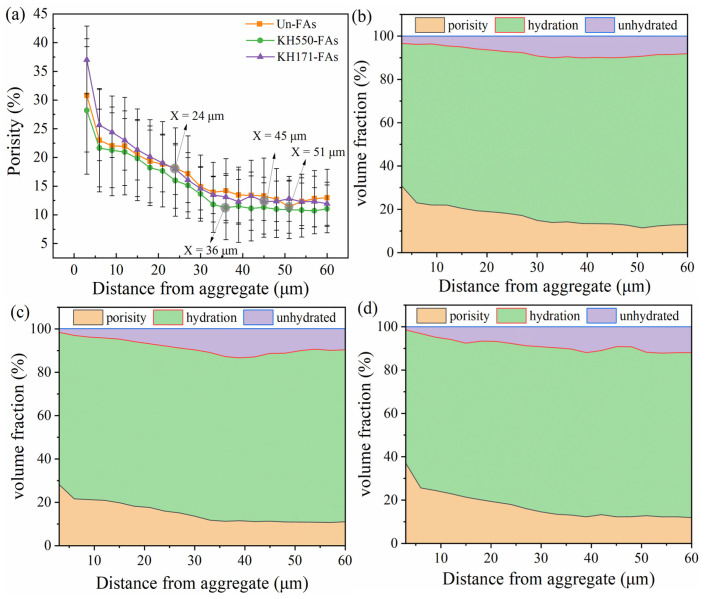
The volume fraction of different minerals against the distance from aggregate: (**a**) porosity; (**b**) Un-FAs; (**c**) KH550-FAs; (**d**) KH171-FAs.

According to the grayscale range of the different phases, a quantitative analysis was conducted on the pores (0–Threshold A), hydration products (Threshold A–Threshold B), and unhydrated cement clinker particles (Threshold B–255) in the ITZ. The results are shown in Figure 12.

The thickness of the ITZ is typically defined as the position where the porosity reaches its final plateau. As observed in Figure 12a, the positions of the gray circles represent the locations where the porosity starts to level off. The results indicate that the ITZ thicknesses for the Un-FAs, KH550-FAs and KH171-FAs are 45 μm, 36 μm and 51 μm, respectively. Compared to the Un-FAs, treating the aggregate with SCAs can reduce the thickness of the ITZ. By examining the variation in porosity in the ITZ, it is found that treating the aggregate with KH550 not only reduces the porosity in the mortar ITZ region but also decreases the porosity in the cementitious matrix. This can be explained by the wettability of the aggregate surface before and after the modification. After modifying the aggregate with KH550, the hydrophilicity of the aggregate surface is reduced, and the ability of the aggregate surface to adsorb water is reduced, so the water film adsorbed on the aggregate surface is relatively thin. This will lead to a reduction in the local water-to-cement ratio of the aggregate surface, thereby reducing the porosity of the ITZ [51]. However, the ITZ porosity of the KH171-FAs increased significantly. Compared with the porosity curve of the Un-FAs, the porosity of the ITZ of the KH171-FAs is higher than that of the Un-FAs in the range of 0–24 μm. This is mainly because after the aggregate is modified by KH171, the aggregate surface is hydrophobic, which makes it difficult for water to spread to the aggregate surface, and thus a large water cavity is formed on the aggregate surface. This makes the local water–cement ratio near the aggregate large, resulting in the enrichment of large crystals of calcium hydroxide and ettringite and an increase in porosity [46]. Figure 12b,c show that the hydration degree of the KH550-FAs was higher. This is mainly due to the reduction in water consumption on the aggregate surface, which allows the cement matrix to obtain more water for cement hydration. By observing the volume fraction of the pores in Figure 12b–d, it is found that the volume fraction away from the aggregate surface is significantly higher than that in other areas and decreases rapidly within 10 μm from the aggregate surface, mainly because the local water–cement ratio is improved by the edge wall effect on the aggregate surface. Moreover, due to the low elastic modulus, the self-shrinkage of the cement slurry is more obvious than that of the aggregate [52].

### 3.10. Nanoindentation

**Figure 13:**
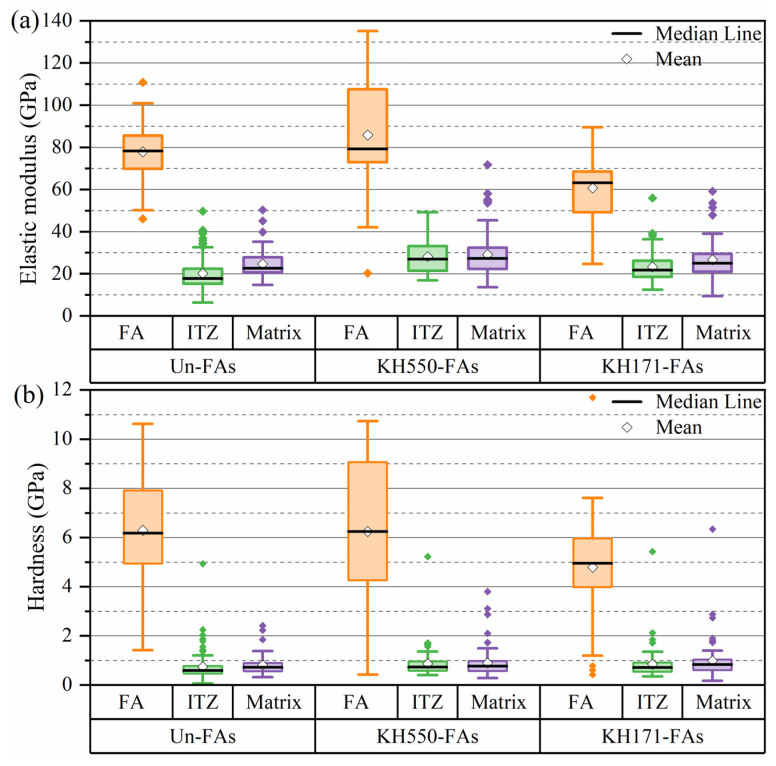
Elastic modulus (**a**) and hardness (**b**) of different regions in mortar before and after aggregate modification.

The micromechanical properties of the ITZ and cementitious matrix were characterized using nanoindentation. Figure 13 presents the average values of the elastic modulus and hardness in different regions of the mortar before and after the aggregate modification.

Figure 13a shows the average values of the elastic modulus in each region of the mortar. It is observed in Figure 13 that the average elastic modulus and average hardness of the ITZ of the KH550-FAs are 28.25 GPa and 0.85 GPa, respectively, which are higher than those of the Un-FAs (20.1 GPa and 0.73 GPa, respectively). Therefore, reducing the aggregate surface wettability has an important effect on the interface’s mechanical properties. Similar to the Un-FAs, the average elastic modulus and average hardness of the ITZ of the KH550-FAs and KH171-FAs are still lower than those of the mortar matrix. This is mainly because the wall effect of the aggregate surface increases the local water–cement ratio of the aggregate surface, resulting in a decrease in strength. It can also be found that the average elastic modulus ratio between the ITZ and the cement matrix of the Un-FAs, KH550-FAs and KH171-FAs is 0.82, 0.97 and 0.88, respectively. Among them, the KH550-FAs exhibit the highest ratio of elastic modulus between the ITZ and the cement matrix, indicating a significant reduction in the wall effect after modification.

### 3.11. Mechanical Properties of Mortar

**Figure 14:**
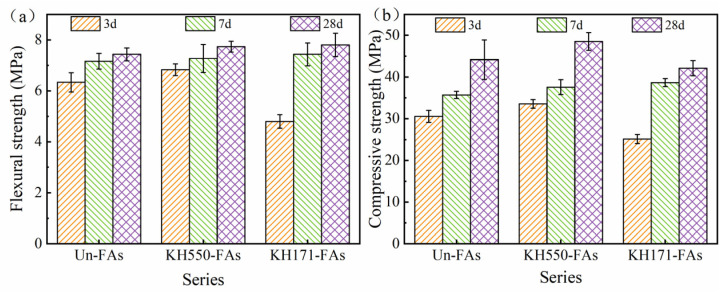
Mechanical properties of mortar: (**a**) flexural strength; (**b**) compressive strength.

Figure 14 shows the mechanical properties of the mortar prepared using an aggregate modification with different types of coupling agents.

The flexural strength and compressive strength of the mortars with different aggregates exhibit varying degrees of development trends. Compared to the Un-FAs, treating aggregates with KH550 improves the flexural strength and compressive strength of the mortar. The flexural strength increases by 1–8% at different ages, while the compressive strength increases by 5–10%. On the other hand, treating aggregates with KH171 has a negative impact on the compressive strength of the mortar, resulting in a decrease of 4–17% at different ages. The results indicate that treating aggregates with KH550 can significantly enhance the mechanical properties of mortar. This is primarily due to two reasons: firstly, treating aggregates with KH550 reduces the interfacial porosity and thickness and enhances the interfacial bond strength and mechanical properties, thereby improving the mechanical performance of the mortar. This is consistent with the conclusion of Xu [38]. Additionally, the improved hydration degree of the cementitious matrix, reduced porosity, and increased elastic modulus indicate that KH550-modified aggregates can densify the cementitious matrix. The combined effect of these factors enhances the mechanical properties of the mortar. However, after treating the aggregates with KH171, the large-scale formation of calcium hydroxide at the ITZ and high porosity result in poor interfacial bond strength, which is the main reason for the decrease in compressive strength [53]. The overall porosity of the KH171-FAs is 26.07%, which is higher than that of the Un-FAs. Porosity disrupts the continuity and uniformity of mortar and becomes the primary site for local stress concentration. Therefore, the high porosity is also a leading factor in the deterioration of the mechanical properties of the KH171-FAs.

## 4. Conclusions

This study investigates the influence of aggregates with different surface wettability on mortar and the interfacial transition zone. Two types of silane coupling agents were used to treat the aggregates, resulting in two different surface wettability conditions. Based on the results, the following conclusions can be drawn.

1. The surface wettability of an aggregate affects the interface bonding property between the aggregate and the cement matrix. The order of the interfacial bond strength of different aggregate surface wettability is 59° > 23° > 91°. Therefore, an appropriate reduction in aggregate surface hydrophilicity can improve the interface bonding property.
2. The mechanism of reducing the surface hydrophilicity of aggregates to improve the interface bonding property is as follows: After modifying the aggregates with KH550, the hydrophilicity of the aggregate surface decreases, resulting in a reduced ability to adsorb water on the surface. This leads to a decrease in the water content on the aggregate surface, which lowers the water-to-cement ratio at the interface and consequently reduces the porosity of the ITZ. The reduction in the water content on the aggregate surface allows the cementitious matrix to obtain more water for cement hydration, thereby enhancing the degree of hydration of the cementitious matrix.
3. The microscopic test results show that the interfacial bonding properties and mechanical properties are enhanced by appropriately reducing the surface hydrophilicity of the aggregates. The variation in the pore volume fraction with a distance from the aggregate surface of 60 μm was quantitatively analyzed with backscattered electron images. The research shows that the hydrophilicity of the aggregate surface can reduce the interfacial porosity and improve the interfacial performance.
4. The surface wettability of the aggregate changes from hydrophilic to hydrophobic, the interface bonding performance deteriorates, and the interface porosity increases. This is mainly due to the poor wettability of the aggregate surface. On the one hand, the addition of hydrophobic aggregates to the cement slurry introduces air bubbles, leading to interface discontinuity. On the other hand, the hydrophobic nature of the aggregates makes it difficult for water to spread onto the aggregate surface, resulting in poor interfacial bonding performance.

## Figures and Tables

**Table 1:** Main chemical composition of cement/wt.%.

Composition	SiO_2_	Al_2_O_3_	Fe_2_O_3_	CaO	MgO	SO_3_	Loss
Mass fraction	20.96	4.13	3.03	62.32	2.90	2.38	2.14

**Table 2:** Basic physical properties of KH550.

Silane Coupling Agent	Molecular Weight	Purity	Melting Point	Boiling Point	Flash Point	Refractive Index	Density (g/cm^3^)
KH-550	221.37	98%	−70 °C	217 °C	96 °C	1.42–1.422	0.95

**Table 3:** Basic physical properties of KH171.

Silane Coupling Agent	Molecular Weight	Purity	Melting Point	Boiling Point	Flash Point	Refractive Index	Density (g/cm^3^)
KH-171	148.23	98%	<−70 °C	123 °C	26 °C	1.392–1.394	0.97

**Table 4:** The mass of the aggregates and the different SCAs.

NO.	Aggregate/g	KH550/g	KH171/g	Ethanol/g	Deionized Water/g
KH550-FAs	1350	0.27	-	2.673	24.057
KH171-FAs	1350	-	1.62	8.461	16.919

**Table 5:** Modification conditions for different SCAs.

NO.	Hydrolysis	Modification	Heating
KH550-FAs	30 °C, 20 min	30 °C, 20 min	100 °C, 3 h
KH171-FAs	30 °C, 2 h	30 °C, 20 min	100 °C, 40 min

**Table 6:** Elemental analysis of the aggregate surface before and after modification.

Elements	Un-FAs/%	KH550-FAs/%	KH171-FAs/%
Si	34.87	33.73	33.6
C	14.65	24.18	31.03
O	50.48	39.35	35.37
N	-	2.74	-

## Data Availability

Data will be made available on request.
